# A data decomposition-based hierarchical classification method for multi-label classification of contractual obligations for the purpose of their governance

**DOI:** 10.1038/s41598-024-63648-x

**Published:** 2024-06-04

**Authors:** Amrita Singh, Preethu Rose Anish, Aparna Verma, Sivanthy Venkatesan, Logamurugan V, Smita Ghaisas

**Affiliations:** 1 Data and Decision Science (DDS) Department, Tata Consultancy Services (TCS) Research, Pune, Maharashtra India; 2 Delivery Excellence Group (DEG), Tata Consultancy Services (TCS), Bangalore, Karnataka India; 3 Delivery Excellence Group (DEG), Tata Consultancy Services (TCS), Chennai, Tamil Nadu India; 4 Ultimatix Department, Tata Consultancy Services (TCS), Chennai, Tamil Nadu India

**Keywords:** Contract governance, Automated extraction, Multi-label classification, Fine-grained governance model, Computer science, Information technology, Software

## Abstract

Contract governance ensures that the agreed outcomes between customers and vendors are fulfilled. Information Technology (IT) outsourcing organizations enter thousands of contractual relationships each month leading to a high volume of business-critical contractual text that must be reviewed and deciphered for effective governance. The key to effective governance of contracts is a model that facilitates assigning ownership of the obligations to the right departments in an organization and allocating their accountability to the right stakeholders. For this, the contractual obligations need to be identified and classified so that details such as actions to be taken by departments in an organization and their ownership as per a given clause are brought out for the purpose of governance. In this paper, we present our work on automated extraction and classification of obligations present in Software Engineering (SE) contracts for the purpose of contracts governance. We propose a novel data decomposition-based hierarchical classification method for a multi-label classification of contractual obligations. We conducted experiments for a fine-grained automated classification of more than 55,000 statements from 50 large real-life SE contract documents received from a large vendor organization into 152 governance-specific classes. The results indicate that the proposed method can bring about a 7–8% improvement in accuracies when compared to state-of-the-art classification baselines such as BERT, RoBERTa, and generative models such as GPT-2.

## Introduction

Contracts provide a foundational framework for commercial and cooperative transactions and relationships. Businesses need to put in place governance mechanisms to ensure that contractual obligations are met, services are delivered as committed, and parties involved in the contract are compliant with all the applicable laws. In the absence of governance, businesses are at risk of suffering reputational, regulatory, and financial damage. The key to an effective governance of contracts is a model that enables assigning ownership of the obligations to the right departments in an organization and allocating their accountability to the right stakeholders. The model should provide a foundation for identifying the obligation(s) embedded in contractual clauses so that these obligations can be extracted and mapped to departments responsible for their fulfilment. To accomplish this, it is not merely enough to extract the obligations. The obligations need to be classified in such a way that details pertaining to the ownership of obligation(s) in a clause are brought out for the purpose of governance. For example, Security Control-related obligations need to be differentiated between physical and information security controls and responsibilities for each need to be placed with the right individuals. Classification at such a fine granularity enables getting detailed insights such as accountability at a granular level and mapping the classified obligations to the right activity so that effective controls are deployed towards fulfilling all aspects of the obligation from the contractual text. The classified obligations are input to a governance system in the vendor organization where they are mapped to the right department/stakeholder and assigned responsibilities as indicated in the classification.

In this paper, we focus on automating the extraction and a governance-focused classification of contractual obligations from Software Engineering (SE) contracts received from a large vendor organization. As indicated above, for the purpose of governance, the classification has to be fine-grained so that accountability with respect to the obligations is ensured. The contracts text however, consists of numerous clauses, terms, and conditions that are susceptible to varying interpretations because of the specialized language—Legalese they employ. The complexity is further exacerbated by cross-references among clauses. This coupled with heavy class imbalance and overlapping linguistic patterns across the classes, make the classification task daunting. To address this problem, we propose a novel data decomposition-based hierarchical classification method for a multi-label classification of contractual obligations. Experiments using different state-of-the-art classification model such as BERT, RoBERTa and generative models such as GPT-2 show that the proposed method can bring about a considerable improvement to the multi-label classification of contractual obligations. To sum up, following are the highlights of our work:

- We propose a fine-grained governance model for the classification of contractual obligations into 152 governance-specific classes using the principles of Grounded Theory (GT).
- We propose a novel method called data decomposition-based hierarchical classification for automating the multi-label classification of contractual obligations into 152 governance-specific classes.
  - To the best of our knowledge, ours is the first work that uses both statements and label specific features in data-decomposition based hierarchical classification method for a multi-label classification of contractual obligations.
  - The results demonstrate that our method performs 7–8% better in terms of accuracy compared to state-of-the-art classification baselines.

## Related work

Abundant work on the application of machine learning and deep learning techniques for automating the extraction or classification of legal text can be found in the literature. In our related work study, we focus only on contracts text.

In previous research endeavors, efforts were made to classify statements of contract. For example, in Indukuri and Krishna^1^, the authors proposed a classification approach to extract clause patterns from e-contract documents. They categorized contract statements as either clauses or non-clauses, and further subclassified clauses as related to payment terms or not. The authors also noted the scarcity of published work on sentence classification in contract documents for the purpose of workflow and monitoring. In Curtotti and Mccreath^2^, the authors proposed an approach that utilized both machine learning and hand-coded methods to classify components of contract texts. In Gao and Singh^3^, the authors introduced a topic modeling-based technique to extract business events and their temporal constraints from contract text. Subsequent studies delved into a finer granularity of contract analysis, where a small set of contract elements, including named entities, were extracted. In Chalkidis and Androutsopoulos^4^, the authors employed deep learning methods to extract various contract elements such as Title, Party, Start, Effective, Termination, Period, Value, Government law, Jurisdiction, Legislative reference, and Headings. In Anish et al.^5^, the authors used Naïve Bayes to classify contractual statements as obligation or non-obligation, while also extracting triggers, actors, time, and action from the identified obligations using rule-based information extraction. In Lee et al.^6^, the authors proposed an automated model for extracting contract-risk to detect “poisonous” clauses in contracts, aimed at supporting contract management for construction companies. Other works^7–12^, similarly focused on the extraction or identification of specific types of contractual statements or clauses, such as obligatory or non-obligatory, ambiguous, or non-ambiguous clauses, contractual risk clauses, and specific contract elements. To the best of our knowledge, our work is the first to concentrate on a fine-grained classification of contractual obligation statements into 152 distinct classes to facilitate contracts governance.

There are works such as^13–17^ that use decomposition-based methods for various multiclass classification problems. We note that none of the existing works tackle multi-label classification problem nor class imbalance problem using data decomposition-based hierarchical classification method on long and complex legal documents such as contracts. Moreover, to our knowledge, this is the first work that uses both statements and label specific features for decomposition-based multi-label classification problem.

### Ethical approval

We took utmost care to ensure that this research was carried out in an ethical and responsible manner. Prior to conducting this research, we obtained all the necessary permissions and approvals from the relevant stakeholders, including the organization that owns the contracts.

## Fine-grained governance model

**Figure 1:**
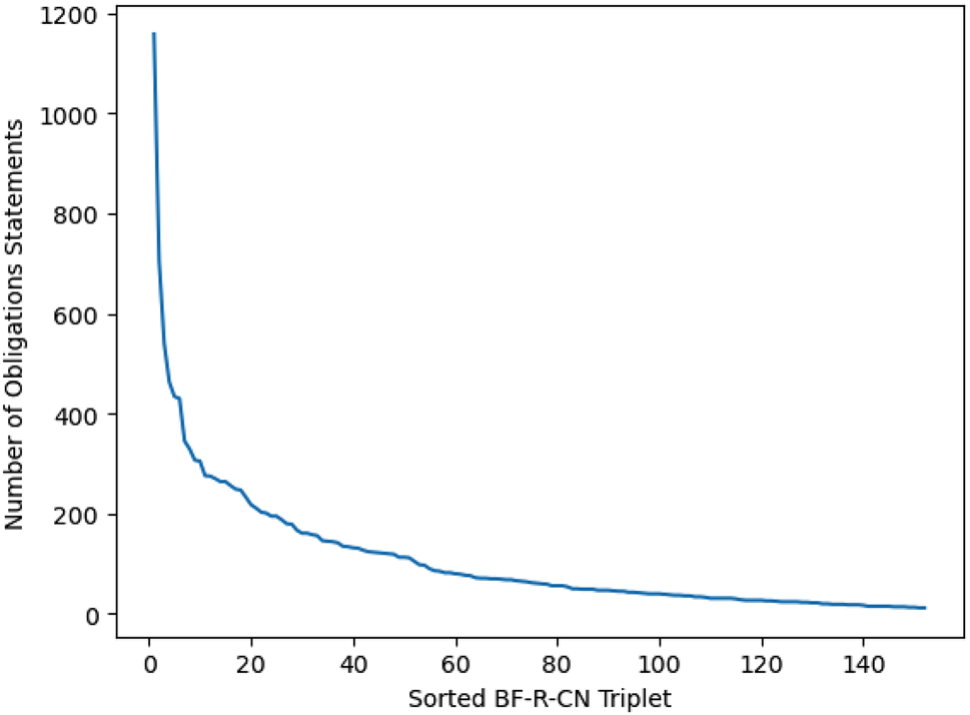
Distribution of obligation statements with respect to BF–R–CN Triplet.

We created the fine-grained governance model by studying 50 Software Engineering (SE) contracts taken from 13 different application domains, including healthcare, automotive, finance, banking, pharmaceuticals, telecom, technology, clothing retail, supermarket, agriculture, e-commerce, manufacturing, logistics and supply chain management. All these contracts are from the same large vendor organization and are executed by distributed teams for multinational companies who have their business activities spread across the globe. The size of each contract document varied between 100 to 500 pages. The contracts were digitized in an HTML format. We used HTML tags present in the documents to identify individual sections and subsequently extracted 57,200 contractual statements (obligations and non-obligations) from these 50 contracts. The obligation statements entail the legal responsibilities and duties that each party is obliged to fulfill as per the terms of the contract. Non-obligation statements include information, definition, or fact statements. The non-obligation statements provide a context but do not need to be governed as opposed to the obligation statements. To create the fine-grained governance model, we followed the guidelines of Charmaz’ Constructive Grounded Theory approach^18^. This approach is commonly employed in social sciences to derive general propositions (referred to as ‘theory’ in this context) from textual data. This method is exploratory and particularly useful in scenarios where researchers do not have pre-existing notions. It is driven by the objective of capturing comprehensive information from data and allowing the theory to naturally emerge. The initial stage involved the first four authors independently reading each contractual statement and assigning coding words to specific portions of the text, such as phrases or paragraphs. These coding words were chosen to signify the relevance of each text segment to a particular aspect of the studied phenomenon. Subsequently, the authors collaborated to systematically reorganize their individually identified concepts into higher-level categories, enhancing the overall understanding of the data. During this process, the authors shared and clarified assumptions, leading to a consensus on the importance of specific concepts and the reasons behind their significance. Similarly, the 57,200 contractual statements were manually examined by the first four authors (referred as annotators) of this paper which included two legal team members from the vendor organization with more than 16 years of experience, also referred to as subject matter experts (SMEs). Initially, two of the annotators (first two authors) classified each statement as an obligation statement or a non-obligation statement. To measure the level of agreement for the multilabel annotations of the two annotators, we utilized the multilabel-kappa Inter-annotator Agreement (IAA) metric. Values ranging from 0.61 to 0.8 for IAA indicate moderate agreement, while a value between 0.8 and 1.0 suggests perfect agreement between two annotators. We obtained a multilabel kappa value of 0.95, which indicates a good agreement in annotation among the two annotators. In cases where there were disagreements among the annotators, the contractual statements were forwarded to the SMEs for deciding the final labels. A total of 16,538 obligations statement and 40,662 non-obligation statements were identified. Since contracts governance calls for a fine-grained obligation classification, each identified obligation statement was further analyzed and manually annotated as a triplet comprising of ***BusinessFunction#Responsibility#CustomerNeed*** (henceforth referred to as ‘BF-R-CN triplet’). The fine-grained classification into triplets was done by the SMEs. Since these SMEs have over 16 years of experience in the domain of contracts governance, their annotations were considered the gold standard. From a contract governance perspective, ***Business Function*** means the department(s) within the organization to which the ownership of an obligation is to be assigned. Some examples of ***Business Function*** are—*Security, Human Resource and Legal*. ***Responsibility*** specifies the duty of the department in fulfilling the obligation. Some examples of ***Responsibility*** are—*Audit, Compliance and Termination*. ***Customer Need*** specifies the details pertinent to *Responsibility* such as *Price Review* in case of *Audit*. Following this nomenclature, the obligation (obfuscated): “*The vendor shall procure its personnel shall comply with all applicable laws and regulations and with customer’s security requirements and policies concerning the handling of sensitive information*” would be annotated as the following triplet: *Security#Compliance#Customer_specific_policy_adherence.* At the end of the manual labeling activity, 152 such triplets were identified. Each of the 16,538 obligation statements present in the dataset was labeled as belonging to one or more of the 152 triplets. 688 obligation statements out of 16,538 statements belonged to more than one triplet. It took the annotators 1080 person hours to label these statements. The global distribution of obligation statements with respect to the sorted 152 BF-R-CN triplets is illustrated in Fig. 1. As can be observed from Fig. 1, there is a long-tailed distribution, indicating that only a few labels had a high number of obligation statements.

Next, we present definitions and examples (with some information obscured) for some of the BF-R-CN triplets that were more prominent in our dataset.

- ***Project delivery-Compliance-Audit:*** This triplet includes clauses that details obligations pertinent to the audit of project delivery processes. For example: *“The vendor shall permit* < *client_name* > *to audit, and perform assurance activities during the Transition Period, to assess and monitor progress against the Transition Plan.”*
- ***Information security-Physical security-Work area restriction:*** This includes clauses that deal with obligations pertinent to physical security related to work areas such as CCTV, asset movement etc. For example: *“The vendor must ensure that communications and server rooms are secured with an access card system.”*
- ***Legal process-Compliance-Law:*** This deals with all the laws that govern the contract and the laws applicable to service delivery. For example: *“Both parties shall ensure that they comply with all reasonable procedures and regulations issued by* < *client_name* > *’s Legal and Compliance Department.”*
- ***Data privacy-Personal data-Access:*** This includes obligations related to access of personal data. For example: *“Vendor shall collect, access, process and transfer Personal Data for the sole purpose of performing Company’s obligations under this Agreement.”*
- ***Onboarding-Compliance-Audit:*** This includes obligations about any audits related to the background checks done during onboarding of resources. For example: *“In the event an audit reveals any issues relating to any background checks, client shall have the right to make copies of such records.”*
- ***Export laws-Export control compliance-Requirements:*** This includes obligations that details the export compliance related requirements. For example*: “To the extent that goods will be transported into the* < *country_name* > *, company represents that it is certified by* < *country_name* > *’s Customs and Border Protection.”*
- ***Vendor IP licensed-New release-Commercials:*** This includes obligations of the vendor with respect to new releases, versions, patches of the product installed in client installation. For example: *“Client will receive minor updates to* < *product_name* > *at no charge and will not be required to implement major updates during the course of this statement of work.”*
- ***Vendor corporate-Finance-Industry standards:*** This includes obligations that list the financial standards mentioned in the contract that vendor as a corporate need to comply with. For example*: “Financial statements should be audited and available, preferably according to recognized accounting standards.”*
- ***Standards-Compliance-Commitments:*** This triplet includes obligations detailing the compliance to audits and standards committed by both the parties involved in the contract. For example: *“Without limiting any of its other obligations under this Agreement, the vendor shall comply with ISO* < *number* > *.”*
- ***People transitions-People takeover-Rehiring:*** This includes obligations that detail the commitments made for people takeover and rehiring. For example: *“Vendor warrants that from the transfer date it will comply with the transfer regulations regarding terms and conditions of employment in respect of the transferring employees.”*

## Automated identification and classification of contractual obligations as per the fine-grained governance model

**Figure 2:**
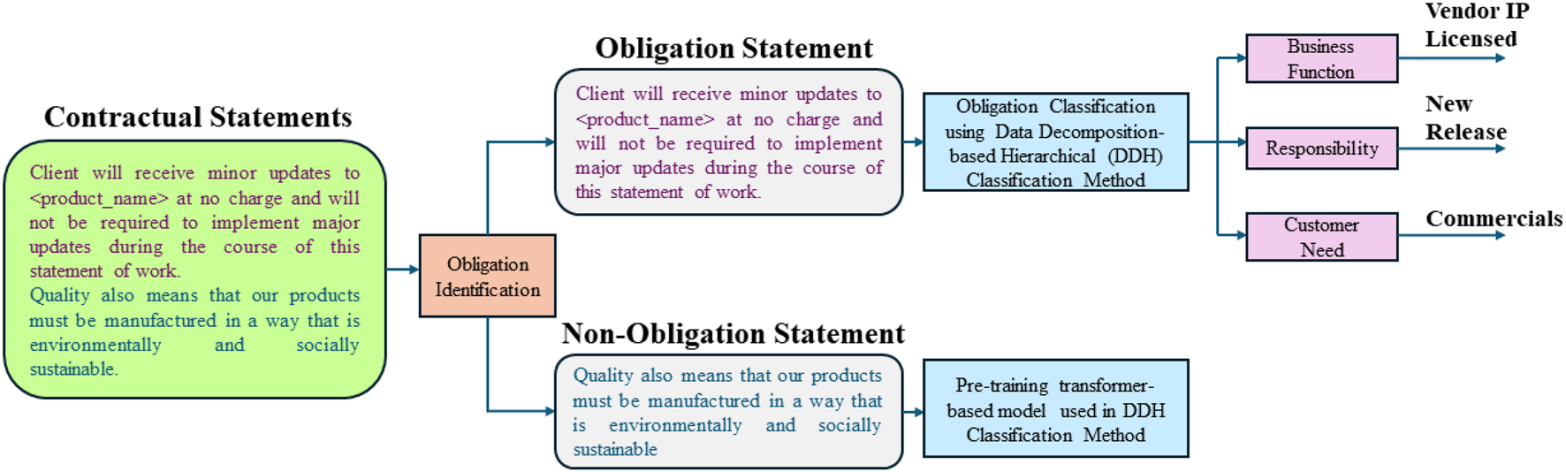
High-level diagram of automated identification and classification of contractual statements.

**Figure 3:**
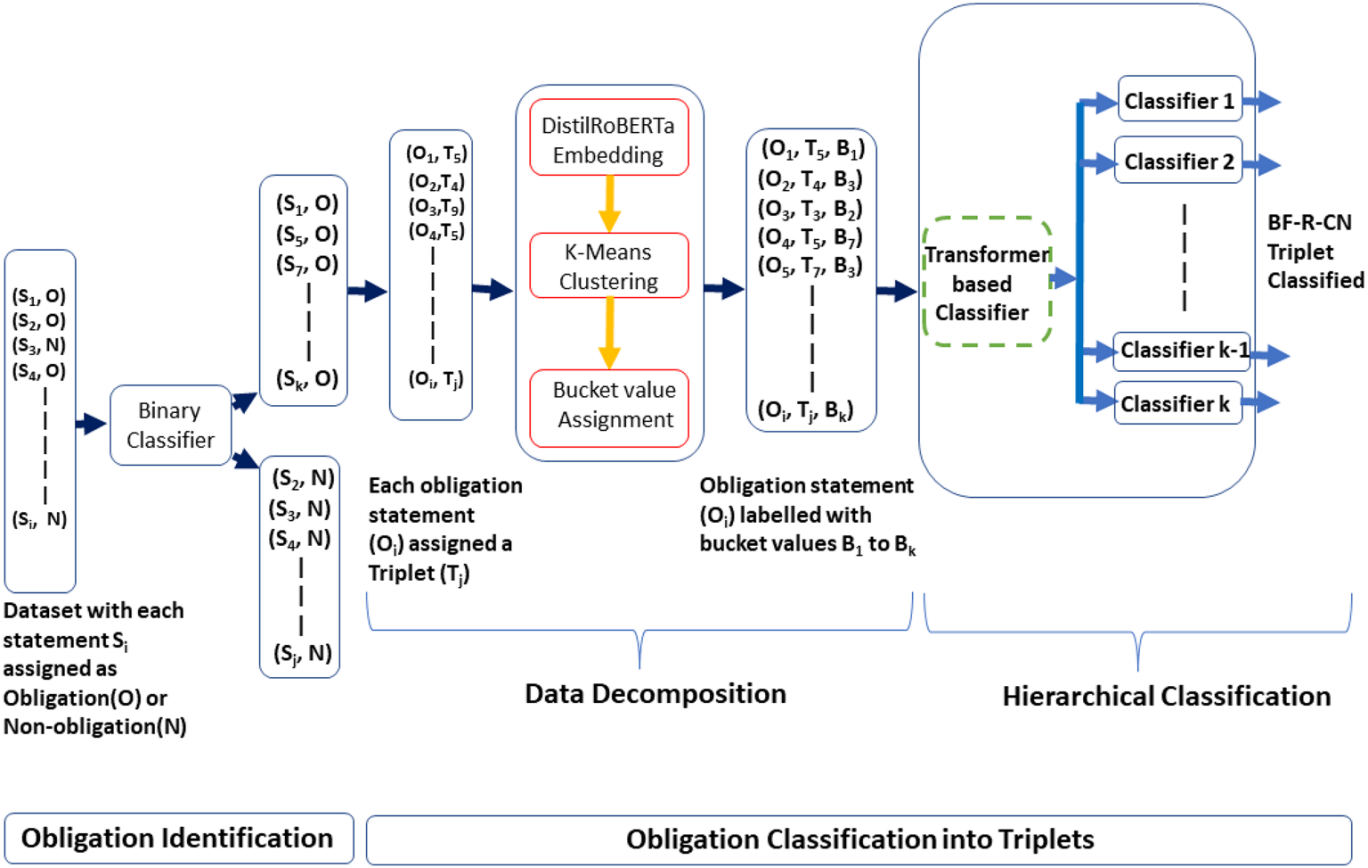
Automated identification and classification of contractual obligations as per the fine-grained governance model.

The labeled dataset detailed in Sect. “Fine-grained governance model” served as the ground truth for automating the identification and classification of contractual obligations as per the fine-grained governance model. Manual labeling of contractual obligations at a fine granularity is an effort-intensive activity. As indicated in Sect. “Fine-grained governance model”, it took the annotators 1080 person hours to label the contractual statements. This indicates the heavy cognitive load associated with manually inspecting the contractual clauses for governance and strengthens the need for automation. We therefore attempted to automate this process. Figure 2 is a high-level diagram depicting the process of automated identification and classification of contractual statements. The resultant labeled dataset however was heavily imbalanced. Some of the triplets such as *Security#Compliance#Customer_specific_policy_adherence* had a high number of instances (1158 instances), whereas some triplets such as *Human_resource#Management#Removal_limit* had low number of instances (14 instances). In Table 1, we present the BF-R-CN Triplet distributions within the dataset for some of the triplets. Figure 3 depicts the detailed approach employed to identify and classify contractual obligations as per the fine-grained governance model.

**Table 1:** Distribution of BF-R-CN triplet.

BF-R-CN triplet	Count
Security#Compliance#Customer_specific_policy_adherence	1158
Data_privacy#Data_privacy_clause#Data_privacy_requirements	706
Delivery#Terminationa_delivery#Termination_right_of_customer_for_cause	540
––	––
––	––
Human_resource#Management#Removal_limit	14
––	––
Delivery#Suspension#Resume_suspended_sow	12

### Obligation identification

To identify obligation statements from contractual clauses, we employed a binary classification approach. Each statement in the dataset was classified as either an obligation statement or a non-obligation statement. The dataset consisted of 57,200 contractual statements, with 16,538 statements manually classified as obligations and the rest as non-obligations. The dataset was preprocessed using standard techniques, including converting the text to lowercase, removing stop words and punctuation, eliminating irrelevant or redundant words, and applying stemming and lemmatization. By reducing noise and redundancy, these preprocessing steps helped the models to focus on the most informative aspects of the text, thus improving their ability to identify obligations accurately. Furthermore, we automatically identified useful bigrams and trigrams for each sentence based on their frequency in the dataset. Bigrams and trigrams that occurred three or more times were added to the corresponding statements. The preprocessed text data was then transformed into numerical features suitable for machine learning algorithms by utilizing Term Frequency-Inverse Document Frequency (TF-IDF).

The traditional machine learning models such as Naive Bayes^19^, Random Forest^20^, and Support Vector Machine (SVM)^21^ were employed in this work, as well as transformer-based architectures. The micro-F1 scores achieved by Naive Bayes, Random Forest, and SVM models were 80%, 90%, and 91%, respectively. Although relatively good performance was demonstrated by the SVM architecture, predictions for certain statements were found to be incorrect due to the presence of numerous keywords and keyword combinations found in other classes, resulting in misclassification. To tackle this problem, BiLSTM with attention^22^ was employed. The results indicated that BiLSTM with attention outperformed other models in identifying obligation statements, achieving a micro-F1 score of 93%, micro-precision of 91%, and micro-recall of 94%. Consequently, the BiLSTM model with attention was selected for identifying obligation statements. For obligation identification, five-fold cross-validation was conducted to train a model with a predefined set of hyperparameter values. The grid search technique was employed to optimize these hyperparameter values. Detailed information regarding the training of each model using various sets of hyperparameters can be found in Table 2.

**Table 2:** Hyper-parameters value of model used in obligation identification.

Model	Hyper-parameters	Value
Complement Naïve Bayes	alpha	0.01
Support Vector Machines	kernel	rbf
	gamma	scale
	class_weight	balanced
	C	10
Random Forests	n_estimator	200
	criterion	gini
	class-weight	balanced
Bi-LSTM	Embedding dimensions	50
	Activation function	Sigmoid
	Bi-LSTM dropout rate	0.5
	Bi-LSTM recurrent dropout rate	0.4
	Bi-LSTM hidden layers	64
	Learning rate	0.002
	Epochs	50
	Sentence length	100
	Optimizer	Adam

### Obligation classification into BF-R-CN triplets using data decomposition-based hierarchical classification method

Once the obligation statements were identified as detailed in Sect. “Obligation identification”, they were classified into one or more triplets using the data decomposition-based hierarchical classification method as discussed below:

#### Data decomposition

To tackle class imbalance, we systematically examined various methods, such as class weights, sample weights, threshold-moving, different weighted-loss functions, tweaking algorithmic knobs, attention mechanisms, and a few ensemble methods. However, the results did not demonstrate improvements compared to our baseline models. Therefore, we considered decomposing the data hierarchically which resulted in getting manageable data chunks. In addition, we posited that a hierarchical approach could offer a way to address the class imbalance by strategically organizing classes into a hierarchy, enabling a more focused classification at each level. Hence, we used the data decomposition technique, wherein the entire dataset was divided into smaller sets referred to as “buckets”. Along with the triplet label, an extra label called bucket value (B1 to Bk) was assigned to each obligation statement in the dataset, indicating which group it belongs to. The process of creating these buckets involves several steps. In the first step, the obligation statements were embedded using the DistilRoBERTa embedding. Next, the embedding was fed into a K-means clustering^23^ model. Finally, the statements were labeled according to the clusters they belong to. The optimal number of clusters (k) was chosen to be 12 through experimenting with different values of k, such as 5, 8, 12, and 15, as detailed in Sect. “Experiments and result”. After forming the clusters, each final label of the statement was assigned a bucket value varying from 0 to 11 based on the cluster with the highest number of statements it contains. The data decomposition method divided the dataset into twelve balanced buckets, each with a comparable number (refer Table 3) of statements.

**Table 3:** Division of the entire dataset into twelve buckets with comparable number of clauses.

Bucket number	Statements distribution
0	975
1	606
2	1055
3	2177
4	2208
5	972
6	1447
7	1543
8	1435
9	1137
10	1689
11	1294

#### Hierarchical classification

We trained a hierarchical classification model with k + 1 transformer-based multi-label classifiers, where k represented the total number of clusters. The model classified obligation statements into triplets. For training, 80% of the total 16,538 statements were used, while the remaining 20% were kept for testing. In the first step, a single multi-label classifier divided each statement in the testing dataset into bucket values ranging from 0 to 11. Then, twelve transformer-based multi-label classifiers were employed in the second step to classify the obligation statements within each bucket into a triplet. Each classifier’s output layer utilized a sigmoid function to normalize the triplet prediction probability, resulting in values between 0 and 1. Testing the model’s sensitivity to different thresholds is crucial to fine-tune its performance to meet requirements effectively. Therefore, the study experimented with different threshold values (0.3, 0.4, 0.5, 0.6, and 0.7). Lowering the threshold value below 0.5 increased the recall but led to more false positives, while raising it boosted precision but resulted in lower recall. Therefore, we found that 0.5 was the optimal threshold value. Obligation statements with predicted triplet probabilities above 0.5 were classified as BF-R-CN triplets.

## Experiments and result

### Comparison with baselines

To compare the data decomposition-based hierarchical classification method against commonly successful techniques, we conducted experiments using three pretrained transformer-based models, namely BERT^24^, RoBERTa^25^, and GPT-2^26^. The selection of transformer-based models for comparison stemmed from their demonstrated efficiency in addressing text classification challenges such as class imbalance and large number of classes in multi-label classification problems. In certain tasks, GPT-2 has exhibited promise in handling complex language structures and comprehending context. Therefore, for our experimentation on multilabel classification of contractual obligations, GPT-2 was included to explore its effectiveness in this domain, alongside more traditional models like BERT and RoBERTa. We abstained from employing other GPT variants such as GPT-3, gpt-3.5-turbo, GPT-4 as we are dealing with confidential and proprietary contracts data and there is a potential risk of disclosure of data when using these GPT variants. The specification of hyperparameters and configuration utilized by transformer-based models in the experiments are given in Table 4. Grid search technique was used to optimize these hyperparameter values.

**Table 4:** The specification of hyperparameter utilized by the transformer-based models BERT, RoBERTa, and GPT-2.

Hyper-parameters	Values
Batch size	32
Sequence length	64
Epoch	100
Activation function	Sigmoid
Loss function	Binary cross-entropy
Optimizer	Adam
Verbose	1
Patience	5
Threshold	0.5
Learning rate	3e−5

### Model pretraining

Several studies demonstrated that pretraining language representation models on large amounts of unlabeled data and finetuning on task-specific data could enhance model performance when compared to training solely on task-specific data^27^. In our experiments, we pretrained all the models, including BERT, RoBERTa, and GPT-2, on 40,662 non-obligatory statements obtained from the 50 contract documents. These non-obligatory statements were similar in style and vocabulary to the obligatory statements, and they also conveyed equivalent semantic information. As a result, these statements could be effectively used to pretrain the language models. To perform pretraining, the data must be in a specific format. The data needs to be structured as a text file (.txt format) with each sentence on a separate line. The purpose of this text file was initially to tokenize the data using either BertTokenizer and RoBERTaTokenizer, and subsequently carry out pretraining on the data. Once the data was converted to the required format, the subsequent step involved training the tokenizer on the input data. This step was helpful in creating the vocabulary for the data. In the next step, we proceeded to pretrain the BERT and RoBERTa model for the masked language modeling (MLM) task. During this process, a specific portion of the input was masked, and instead of predicting the following word, the network focused on predicting the masked tokens. This training strategy played a critical role in achieving bidirectional conditioning and enabled the model to grasp the connection between masked words and their surrounding left and right context. To accomplish this, we utilized the 40,662 non-obligation statements, and employed them to train the tokenizer for this purpose. For the MLM task, 15% of the tokens were randomly masked, and the model was then trained to predict those masked tokens. We employed the grid search technique to optimize the hyperparameter values. The hyperparameters employed for the pretraining of BERT, RoBERTa, and GPT-2 are described in Table 5. BERT and RoBERTa were trained on the MLM task, while GPT-2 was trained using a left-to-right autoregressive approach. GPT-instead,ot employ MLM like BERT and RoBERTa and instead is trained by predicting the next word in a sequence given the preceding words using a left-to-right approach.

**Table 5:** Hyper-parameters for Pretraining BERT, RoBERTa & GPT-2.

Model	Hyper-parameters	Values
BERT & RoBERTa	block_size	512
	mlm_probability	0.15
	num_train_epoch	100
	per_device_train_batch_size	24
	per_device_eval_batch_size	24
	learning_rate	5e-5
GPT-2	per_device_train_batch_size	3
	per_device_eval_batch_size	3
	evaluation_strategy	“steps”
	logging_steps	5_000
	eval_steps	5_000
	gradient_accumulation_steps	8
	num_train_epochs	20
	weight_decay	0.1
	warmup_steps	1_000
	lr_scheduler_type	“cosine”
	learning_rate	5e-4
	save_steps	5_000
	context_length	1024
	total_epochs	100

### Evaluation metrics

For evaluation of the experiments, we employed micro-F1-Score. This requires computing micro-Precision, and micro-Recall beforehand, as micro F1-score is the harmonic mean of micro-averaging precision and recall. The advantage of utilizing micro F1-Score lies in its capacity to alleviate the impacts of class imbalance and its effectiveness in handling multilabel classification by treating all classes equally and considering precision and recall across the entire dataset. Below is the formula for all three metrics used in the experiments:

$${\text{micro-Precision}}\left( {\text{P}} \right) = \left( {\sum\nolimits_{\text{i=1}}^{{\text{N}}} {{\text{TP}}_{{\text{i}}} } } \right)/\left( {\sum\nolimits_{{{\text{i}} = 1}}^{{\text{N}}} {{\text{Predicted}}\;{\text{Positives}}_{{\text{i}}} } } \right)$$

where N is the total number of classes, TP_i_ is the true positive for class i, Predicted Positives_i_ ​ is the sum of predicted positives for class i.

$${\text{micro-Recall }}\left( {\text{R}} \right) = \left( {\sum\nolimits_{\text{i=1}}^{{\text{N}}}{{\text{TP}}_{{\text{i}}} } } \right)/\left( {\sum\nolimits_{\text{i=1}}^{{\text{N}}}{{\text{Actual}}\;{\text{Positives}}_{{\text{i}}} } } \right)$$

where N is the total number of classes, TP_i_ is the true positive for class i, Actual Positives_i_ ​ is the sum of actual positives for class i.

$${\text{micro}}\;{\text{F1-score}} = \left( {{2}*{\text{P}}*{\text{R}}} \right)/\left( {{\text{P}} + {\text{R}}} \right)$$

where P is micro-Precision and R is micro-Recall.

### Results and discussion

In Table 6 we present the results obtained applying the proposed data decomposition-based hierarchical classification method on 3 different models and the baselines in terms of micro-Precision (P), micro-Recall (R) and micro F1- score (F1). The proposed method applied on three different models demonstrates superior performance in classifying obligation statements when compared to the baselines.

**Table 6:** Comparing the result of the proposed data decomposition-based hierarchical classification method (DDH) using different models and Baselines.

Model	P	R	F1
BERT	0.64	0.55	0.59
RoBERTa	0.67	0.58	0.62
GPT-2	0.63	0.4	0.49
DDH + BERT	**0.79**	**0.58**	**0.67**
DDH + RoBERTa	**0.83**	**0.59**	**0.69**
DDH + GPT-2	**0.76**	**0.45**	**0.57**

Significant values are in bold.

The baseline models such as GPT-2 lacked precision in generating fine-grained predictions for closely related classes, leading to accuracy issues. Moreover, in specialized domains, where contextual factors such as legal jargons and complex relationships are crucial, GPT-2 model’s text generation approach was less effective when compared to models such as RoBERTa and BERT, designed specifically for text classification. From Table 6 it is evident that DDH + RoBERTa achieved the highest micro F1-score of 69% in classifying the obligation statements into 152 triplets. Furthermore, the results reveal that by employing the proposed method on three different models (DDH + BERT, DDH + RoBERTa, DDH + GPT-2), we are getting a consistent 7–8% improvement in accuracy, in comparison to state-of-the-art classification baselines such as BERT, RoBERTa, and generative models such as GPT-2.

Specifically, when using the proposed method, DDH + GPT-2 shows a 13% improvement in precision and a 5% improvement in recall. DDH + BERT demonstrates a 15% improvement in precision and a 3% increase in recall. DDH + RoBERTa exhibits a 16% improvement in precision and a 1% improvement in recall. This indicates the efficacy of the proposed method in addressing the challenge of low precision and recall observed in the baselines.

The contracts dataset contains contractual clauses with complex “Legalese” and cross-references, making the classification task very challenging. The dataset also suffers from heavy class imbalance, with some classes being dominant while others are minority. This led to poor performance when baseline classifiers such as BERT, RoBERTa, and GPT-2 were used. By decomposing the dataset into clusters, we simplify the complexity and address the class imbalance inherent in contracts dataset. The decomposition enables the classifier to operate on more focused and manageable subsets of the data, improving its ability to discern patterns and make accurate classifications. For instance, consider the obligation statement “*Availability of new servers, Non-Prod and Prod ICP instances across environments with required capacity has to be ensured by the Bank*.” Its final label is ‘Delivery#Environment#Access.’ However, baseline models such as RoBERTa incorrectly predicted additional labels such as ‘Security#Infrastructure#Specific_infrastructure’ for the above obligation. Clustering helps the classifier focus on relevant subsets, reducing such misinterpretations thereby improving classification accuracy. Furthermore, the hierarchical organization of the clusters allows us to tackle class imbalance at different levels, thereby mitigating the effects of global class imbalances. By implementing a method that enhances the accuracy of obligation classification, we contribute towards streamlining the contracts governance process by reducing the time and resources spent on manual classification.

### Ablation study

In the proposed data decomposition-based hierarchical classification method, the determination of the optimal value of clusters (k) was crucial for achieving acceptable accuracy. To determine the optimal value of k, experiments were performed with different values of k, while varying the sequence length and batch size. A study was conducted to analyze the results of these experiments, enabling us to determine the impact of each chosen value of k on the overall accuracy of the pipeline. The results presented in Table 7 indicate that the optimal value of k was found to be 12. Beyond this value, a saturation point was reached, and further increase in the value of k did not improve accuracy. The highest micro F1-score was observed when k was set to 12. It was also observed that the division of the dataset into twelve buckets, as presented in Table 3, resulted in a more evenly distributed dataset, thereby resolving the issue of data imbalance. Thus, the optimal value of k was selected as 12.

**Table 7:** Result of ablation study.

Setting	K	P	R	F1
Batch size = 16 Seq length = 490	5	0.63	0.58	0.6039
	8	0.80	0.61	0.6921
	**12**	**0.82**	**0.6**	**0.6929**
	15	0.82	0.6	0.6929
Batch Size = 32 Seq length = 64	5	0.76	0.55	0.6381
	8	0.82	0.57	0.6725
	**12**	**0.83**	**0.59**	**0.6897**
	15	0.83	0.59	0.6897

Significant values are in bold.

Next, we conducted multiple experiments to find the optimal setting for our experiments. The results presented in Table 8 indicates that a 12 × improvement in training efficiency was achieved by setting the average sequence length to 64 and the batch size to 32, compared to the scenario where the maximum sequence length was set to 490 and the batch size was set to 16. Optimizing the sequence length and batch size at 64 and 32, respectively, maximized precision, with a minor decrease in recall of approximately 1% when k was set to 12. Therefore, the optimal configuration for the experiment was determined to be a batch size of 32 and a sequence length of 64.

**Table 8:** Runtime of each model on different settings. Training time was measured and compared in seconds per epoch.

Setting	Model	Training time (s)
Batch size = 16 Seq length = 490	BERT	165
	RoBERTa	167
	GPT-2	178
Batch size = 32 Seq length = 64	BERT	13
	RoBERTa	14
	GPT-2	14

## Discussion and conclusion

**Figure 4:**
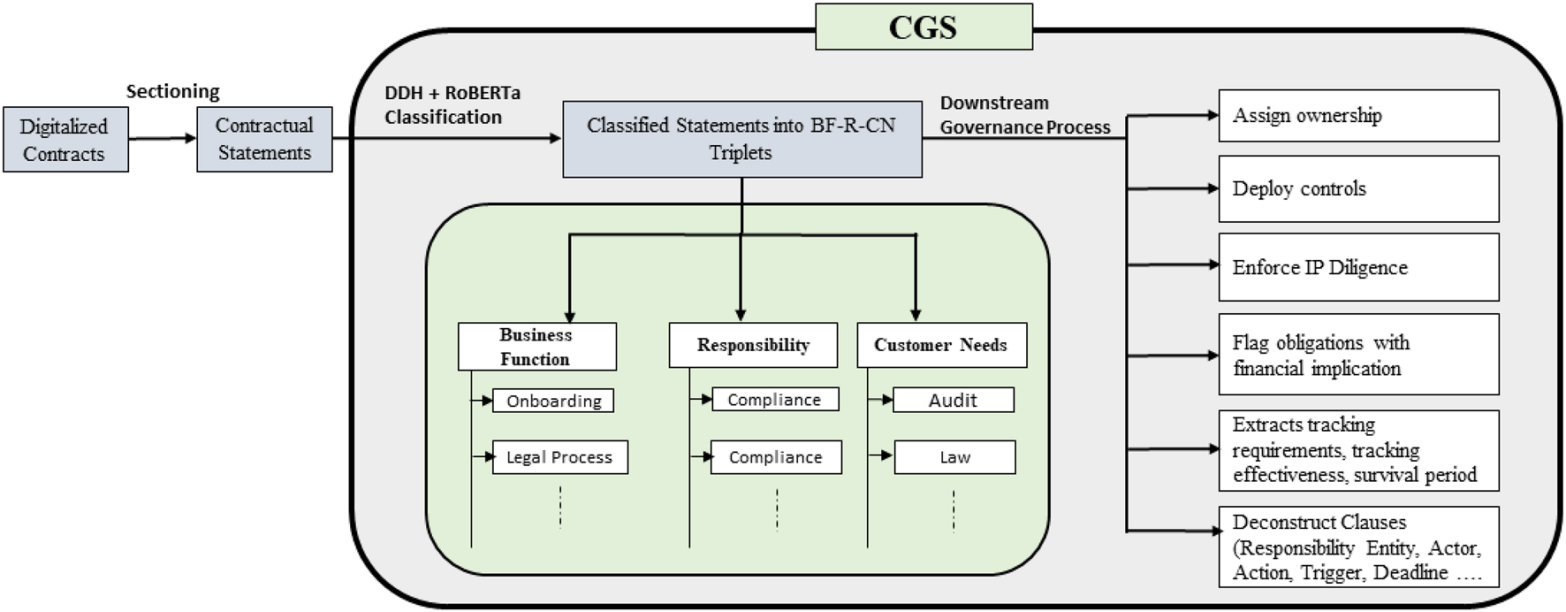
Contracts governance system (CGS).

In this paper we report a fine-grained governance model for classification of contractual obligations into 152 governance-specific classes using the principles of Grounded Theory (GT). We further report the automation of the identification and classification of contractual obligations. The work utilizes real-life contracts documents from a large IT outsourcing vendor organization that executes projects from across the globe. We propose a novel data decomposition-based hierarchical classification method that uses both statements and label-specific features for multi-label classification of contractual obligations. The results of experiments conducted on classification of 16,538 obligation statements from 50 large contract documents into 152 governance-specific classes indicate that the proposed method significantly outperforms the baselines models. The proposed method addresses the challenges posed by complex multi-label classification tasks, particularly those involving a large number of classes and heavy class imbalance. By implementing a method that enhances the accuracy of obligation classification, we attempt to streamline the contract governance process by reducing the time and resources spent on manual review and interpretation, and save costs associated with human error and inefficiency. To the best of our knowledge, the proposed method is the first of its kind designed specifically for classifying contractual obligations into many classes (152). The proposed method will be a part of the Contracts Governance System (CGS) that is deployed in the vendor organization. Figure 4 pictorially represents CGS. Next, we briefly explain CGS.

CGS provides a foundation for understanding the obligations and ensuring that the information vital to the success of a software project gets extracted and highlighted to the right stakeholders. Further, CGS assigns an obligation to respective owner(s) and maps it to the right activity so that effective controls are deployed towards fulfilling all aspects of the obligations. Once the obligations are automatically identified and classified, they are further processed to support downstream governance processes. The downstream governance process includes extraction of critical elements from the classified obligations such as Responsible Entity, Action, Trigger, Deadline. CGS also flags obligations with financial implications such as milestones with penalties to increase rigor and compliance. Further, for each obligation, CGS extracts its tracking requirements, tracking effectiveness, survival period of obligations and ownership for fulfilment. CGS currently tracks and governs 100% of contracts (30,000 + active contracts) signed between the vendor organization and several multinational companies across the globe. As per the contract governance team in the vendor organization, the projected savings on full deployment of the proposed data decomposition-based hierarchical classification method across the organization in near future is expected to be ~ 60%.

## Limitations and future work

The proposed Data Decomposition-based hierarchical classification method leveraged a two-level hierarchical model. This required multiple models to classify their respective buckets of target classes, thereby increasing the storage requirements on the deployment server. We plan to mitigate this challenge by exploring solutions such as the use of adapter modules^28^. Furthermore, over time, there is a possibility of an influx of new contracts that exhibit different linguistic patterns than the ones on which the model is trained. Retraining the models in such scenarios may be required. However, we believe that the classes would saturate over a period thereby mitigating this limitation.

Currently, we have created the fine-grained governance model by studying 50 Software Engineering (SE) contracts taken from 13 different application domains, including healthcare, automotive, finance, banking, pharmaceuticals, telecom, technology, clothing retail, supermarkets, agriculture, e-commerce, manufacturing, logistics, and supply chain management. As future work, we plan to train the model further by including contract documents from more diverse domains such as embedded systems, aerospace etc. This step aims to enhance the generalizability of the proposed approach to such diverse domains. Furthermore, we plan to establish a feedback loop involving experts from the contract governance domain. By collecting feedback on misclassifications, the model can be continuously improved thus supporting adaptability. Additionally, we plan to explore other techniques such as knowledge distillation, pruning, and quantization, which can help reduce the computational requirements without a significant loss of performance.

In future, we plan to add a rationale generation module to the classified obligations. Understanding why a particular classification decision is made will bring in transparency and thereby increase trust in the model generated output. We also plan to incorporate a summarization module that will take the classified contractual clauses and condense them into concise summaries. These summaries will provide an overview of the triplets, making it easier for the stakeholders to grasp the essential elements of the contractual clauses without delving into the full text. Additionally, we will continue to focus on the scalability and adaptability of the proposed system. As more contracts and variations are encountered, we intend to refine the fine-grained governance model to accommodate a broader range of contract types, languages, and an evolving legal terminology.

## Data Availability

The datasets generated and/or analyzed during the current study are not publicly available due to the highly confidential nature of the real-life contractual data used. Our primary concern is to protect the confidentiality of the data and comply with contractual agreements. Disclosure of this data would result in a breach of contract and legal consequences for the parties involved, including the organizations entrusting us with the data. Therefore, we are unable to release the contracts dataset but are available from the corresponding author on reasonable request.
